# A New Species of the Genus *Parasa* Moore (Lepidoptera: Limacodidae) from Yemen

**DOI:** 10.1673/031.010.19001

**Published:** 2010-11-03

**Authors:** Alexey V. Solovyev, Aidas Saldaitis

**Affiliations:** ^1^ Zoology Department, Ulyanovsk State Pedagogical University, Ulyanovsk, Russia; ^2^ institute of Ecology of Vilnius University, Vilnius, Lithuania

**Keywords:** *Parasa dusii*

## Abstract

A new species *Parasa dusii* Solovyev and Saldaitis from northern Yemen is described (holotype in Museum Witt, Munich; Germany). The species has tendency to lose the green pigment typical for other congeners. It is provisionally placed into the genus *Parasa* Moore, 1859 where it is closely related to *P. divisa* West, 1940, *P. catori* Bethune-Baker, 1911, *P. marginata* West, 1940, *P. thamia* Rungs, 1951, *P. dentina* Hering, 1932, *P. ananii* Karsch, 1896, and *P. semiochracea* Hering, 1933. The relationship of the new species to these African species suggests its secondary penetration into the Arabian Peninsula from an origin in tropical Africa. The problems of monophyly of the genus *Parasa* and several associated genera are briefly discussed. All important characters of the new species, and some related species, are illustrated.

## Introduction

A new species of the genus *Parasa* Moore, 1859 is described that was found in northern Yemen by the junior author. This unique species is very interesting in its relationships and zoogeography. The species is quite different in wing coloration as the typical green pigment is often changed to reddish yellow, and the forewing pattern and male genitalia are close to some African species of *Parasa.*

At present, the genus *Parasa* Moore, 1859 includes about 250 species worldwide and ranges mainly in the tropics and subtropics. Its monophyly is not evident, and the species from America, Asia or Africa are rather different, with the presence of distinct groups of species endemic to each region. Perhaps, the distribution of the genus *Parasa* auct. is limited to America, and in other regions the green species has evolved in other phylogenetic lineages. The revision of Asian ““Parasoid”” species is in preparation, and so far, close generic relationship with American species has not been found. The African species of this complex includes about 100 described species and at least five easily recognized groups that include, respectively, the type species of the names *Latoia* GuéérinMééneville, 1844, *Stroter* Karsch, 1899, *Delorhachis* Karsch, 1896, *Lemuriostroter* Hering, 1957, and *Letois* Felder, 1874. Weak diagnoses of these genera have caused the misplacing of a large number of species. Sometimes several genera of the mentioned are regarded as synonyms (Eecke, 1925; Holloway, 1986; Freina & Witt, 1990) but it is not confirmed here. A comprehensive revision is needed, and the present paper makes some steps toward achieving it.

A new species is provisionally placed into the genus *Parasa* that is in accordance with the very broad but questionable concept of the genus *Parasa.* The exact generic placement of the new species will be possible after revision of *Parasa* worldwide. Its generic diagnosis is not clear and needs further investigation and testing.

## Material and Methods

The specimens were collected using standard methods with a black light bulb.

The male genitalia were examined using standard methods. The apex of the abdomen was macerated in 10% solution of alkali in a water bath for 10 minutes. The genitalia were dissected using micro-forceps; the aedeagus was separated and stained with Evans blue dye for 5 minutes for staining of vesica. The genitalia were then mounted in Euparal and labeled.

Digital images were made using a Nikon (www.nikon.com) Coolpix 5400 camera and a binocular microscope MBS-9. The images were improved and prepared for publication using Corel Draw 13 and Corel Photo-Paint 13 (www.corel.com).

## Description

### *Parasa dusii* Solovyev and Saldaitis, new species

***Male*:** (Figures 1, 2, 3) The moths are robust, medium-sized with forewing length 10.5–12.0 mm and wingspan 24.0–25.0 mm. The head is green or brown. The antennae are brown, broadly bipectinate in basal quarter and almost filiform distally, with total length of 2/3 forewing. The labial palps are covered by brown scales, with yellow terminal segment; their 2^nd^ segment is medium-sized, as long as a half of the eye diameter; the 3^rd^ segment is short, as long as one third of the 2^nd^ one (Figure 3, ““*P.lab II”” &* ““*P.lab III””*). The thorax is green or reddish yellow, like the medial part of the forewing. The forewings are elongated, with concave costa, brown, with large green or reddish yellow medial area that reaches the costa and dorsum, with dark brown basal part and paler distal one. The distal border of this medial area is dark brown, sinuous, running from 2/3 costa to 2/3 dorsum, with characteristic strong curvature near the costa. The border of the medial and brown basal areas, running from 1/3 costa to 1/3 dorsum, is clearly defined. The hindwings are pale yellow with grizzly grey tint. In the forewing, the vein R1 is not sinuous; the vein R5 is branched from the basal part of the branch R3+R4; the medial stem is dichotomous divided over its distal third; the veins M2 and M3 are well separated basally. The hindwing venation is typical for the Limacodidae (Janse, 1964; Holloway, 1986), the veins Sc+R1 and Rs are anastomosed basally. The abdomen is pale yellow, with a brown basal spot dorsally. The tibial spurs formula is 0-2-2.

***Male genitalia*:** (Figures 4, 5) The uncus is triangular in back view, laterally haired, apically slightly flattened, strongly sclerotized and claw-shaped. The gnathos is large, wide, strongly sclerotized, narrowed distally, as long as 2.2–2.5 of uncus length, and curved up in the proximal third. The valvae are elongated, narrow, triangular-shaped with concave costa, with clearly defined, strongly sclerotized sacculus and weak, very narrow cucullus; hemitranstilla is developed. The juxta is flattened, medially with a dorsal rounded incision. The saccus is indistinct. The aedeagus is tube-shaped, as long as 1.3 of valva length, and curved basally with distinct coecum. The vesica is without cornuti.

***Female and immature stages*:** Unknown.

***Holotype*:** ♂?, ““N. Yemen, || Al Hudaydah prov., || Wadi Bura, || 9.01.2010. || Saldaitis leg.””, MWM,

***Paratypes*:** 1♂?, ““N. Yemen, Al || Mahwit prov., || Wadi Sara || 27.03.2009. || Saldaitis leg.””, CASV. 2♂?♂?, ““N. Yemen, Al Hudaydah prov., || Wadi Bura || 28.03.2009. || Saldaitis leg.””, CASU & CASV.

***Diagnosis*:** The species is similar externally to *Parasa divisa* West, 1940 (TL: Ghana), *P. catori* Bethune-Baker, 1911 (TL: Nigeria), *P. marginata* West, 1940 (TL: Angola), *P. thamia* Rungs, 1951 (TL: Morocco), *P. dentina* Hering, 1932 (TL: Nigeria), *P. ananii* Karsch, 1896 (TL: Togo), and *P. semiochracea* Hering, 1933 (TL: Sierra Leone) (Figures 6–9, 12–15) but the distal brown forewing area of the new species is bounded by a dark brown facia, sinuous, with strong curvature near the costa, running from 2/3 costa to 2/3 dorsum; the medial forewing area is green or reddish yellow; the thorax is green or reddish yellow. The male genitalia of new species differ from mentioned species by the narrow, not curved up distally valvae.

**Figure 18.**
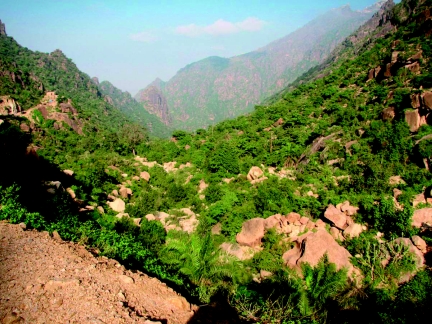
The biotope of Wadi Bura (Yemen), a locality of the paratypes (from Wadi Bura) of *Parasa dusii* sp. n. High quality figures are available online.

***Biology*:** The specimens were collected in early January and late March in Wadi Bura (600–700 m) and Wadi Sara (1200 m) valleys situated on the western slopes of Asir Mountains facing savannah type Al Hudaydah plane and Red Sea. Wadi Burra and Wadi Sara are in the Jebel Burra mountains area where minor subtropical rainforests with rich flora of different plant associations are still left (Figure 18).

***Etymology*:** The species is named after Stefano Dusi (Italy) for his contributions to entomology.

## Discussion

A new species of the genus *Parasa* Moore, 1859 is described. Its exact systematic position is unclear because of a necessity of the revision of the African *Parasa.* The new species is untypical, with unusual tendency of substitution of the green pigment that is typical for other *Parasa* by reddish yellow.

**Plate 1.**
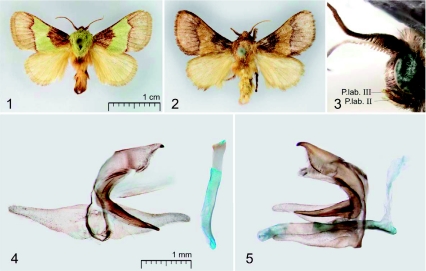
**Figures 1–5.** *Parasa dusii* sp. n.; **1** —— Male holotype; **2** —— Male paratype (from Wadi Bura); **3** —— head of the paratype (from Wadi Bura), lateral view; **4** —— male genitalia of the holotype, aedeagus separated, back view; **5** —— male genitalia of the paratype (from Wadi Bura), lateral view. Abbreviation: ““P.lab. II”” and ““P.lab. III”” means the second and third segments of labial palps correspondingly. High quality figures are available online.

**Plate 2.**
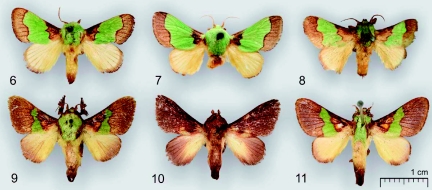
Figures 6–11. External view of the related species; **6** —— *Parasa ananii* Karsch, 1896, holotype, ZMHB; **7** —— *P. thamia* Rungs, 1951, holotype, MNHN; **8** —— *P. catori* Bethune-Baker, 1911, holotype, BMNH; **9** —— *P. divisa* West, 1940, holotype, BMNH; **10** —— *Delorhachis amator* Hering, 1928, holotype, ZMHB; **11** —— *Stroter comatus* Karsch, 1899, holotype, ZMHB. High quality figures are available online.

The specimens with both types of coloration are included in the type-series. Cases of such substitution of the green pigment by yellow or brown are known also for other Limacodidae and may cause description of numerous synonyms. For example, three related species, *Latoia albicosta* (Hampson, 1910), *L. intermissa* (Walker, 1865) and *L. anagaura* Janse, 1964 (generic combinations are given provisionally) from southern Africa are probable synonyms and characterized by the same forewing pattern but the colorations are different. The species *L. intermissa* has green pigment on the thorax, head and forewings, but this pigment is substituted by ochre in *L. albicosta* and *L. anagaura.* The male genitalia of all three species are rather similar and hardly distinguishable, with a very long aedeagus bearing an apical incision and two small spurs, very broad gnathos and apically prominent juxta with tape-like process underlying the aedeagus. Such colour substitution can be related to ecological factors or development of reproductive isolation from closely related species involving recognition of the species. These hypotheses need further investigation.

**Plate 3.**
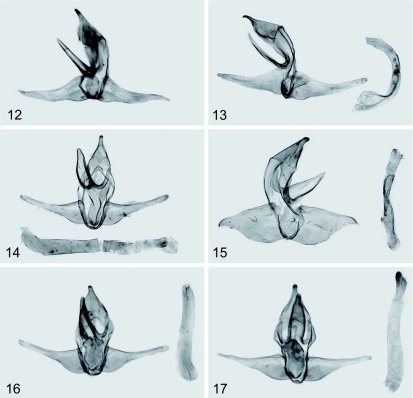
Figures 12–17. Male genitalia of the related species; **12** —— *Parasa ananii* Karsch, 1896, holotype, ZMHB (aedeagus is not imagined here); **13** —— *P. catori* Bethune-Baker, 1911, holotype, BMNH; **14** —— *P. divisa* West, 1940, holotype, BMNH; **15** —— *P. marginata* West, 1940, holotype, BMNH; **16** —— *Delorhachis amator* Hering, 1928, holotype, ZMHB; **17** —— *Stroter comatus* Karsch, 1899., holotype, ZMHB. High quality figures are available online.

Taking into account the forewing pattern and the features of the male genitalia, the species *dusii* sp. n. may be closed to *Parasa divisa* West, 1940, *P. catori* Bethune-Baker, 1911, *P. marginata* West, 1940, *P. thamia* Rungs, 1951, *P. dentina* Hering, 1932, *P. ananii* Karsch, 1896, *P. semiochracea* Hering, 1933, *Delorhachis amator* Hering, 1928, and *Stroter comatus* Karsch, 1899. Some of these species and their genitalia are illustrated for comparison (Figures 6–11, 12–17). The species *amator* (Figures 10, 16) has reduced forewing pattern with a small yellow spot near 1/3 dorsum; currently it is mistakenly regarded as a member of the genus *Delorhachis* Karsch, 1896 and its exact systematic position needs investigation. All species listed, including the new one, are characterized by a complex of the following diagnostic features. The male antennae are broadly bipectinate in basal quarter, simple distally. The forewings are elongated, with concave costa. The forewing pattern consists of a basal brown area, pale brown distal area, and a medial area of any distinct color (green or different tints of yellow). The male genitalia are mostly diagnostic, with the uncus triangular in shape when viewed dorsally, lacking any spur; the gnathos is wide, strong and narrowed distally. The valvae are without saccular processes but with distinct sacculus and narrow weak cucullus. The juxta is unmodified, flattened. The aedeagus is tube-shaped, without any apical process. The vesica is without cornuti. The relationship of all mentioned species is probable, but no unique features have been found so far except this complex of diagnostic characters. As stated above, the species are regarded as members of different genera in spite of their close relationship. Their generic combinations are still provisionary until a revision of the ““Parasoid”” complex is made and the diagnoses of all involved genera are improved.

It seems to be likely that *P. dusii* sp. n. penetrated the southern Arabian Peninsula from continental Africa where closely allied species are distributed. The new species was collected in Wadi Bura and Wadi Sara in wet subtropical forests similar to those of eastern Africa. The presence of the species here shows that the south-west of the Arabian Peninsula has strong influence from the Ethiopian Zoogeographic region. A similar invasion to the north from southern and central Africa is known for a related species from the ““parasoid”” complex, *Parasa thamia* Rungs, 1951, known from north-eastern Morocco and is considered to belong to the Palaearctic fauna (de Freina & Witt, 1990: 43). It is also known for the south-western Palaearctic *Coenobasis farouki* Wiltshire, 1947, ranges to Sinai [Egypt] and southern Oman (Wiltshire, 1990); the remaining 9 congeners of *Coenobasis* Felder, 1874 are found only in the Ethiopian region (Hering, 1928; Janse, 1964). In northern Oman the limacodid species *Deltoptera omana* Wiltshire, 1976 is also known and its relation to South African region is discussed in Wiltshire (1990).

## Abbreviations

**BMNH**,: the Natural History Museum - London, United Kingdom;
**CASU**,: collection of Alexey Solovyev - Ulyanovsk, Russia;
**CASV**,: collection of Aidas Saldaitis - Vilnius, Lithuania;
**MNHN**;: Musééum National d'Histoire Naturelle - Paris, France;
**MWM**,: Museum Witt, Munich - Germany;
**TL**,: type locality;
**ZMHB**,: Zoologisches Museum der Humboldt Universitäät zu Berlin - Germany;
**““|””**,: a new line in the citations of labels
